# A combination intervention addressing sexual risk-taking behaviors among vulnerable women in Uganda: study protocol for a cluster randomized clinical trial

**DOI:** 10.1186/s12905-019-0807-1

**Published:** 2019-08-17

**Authors:** Fred M. Ssewamala, Ozge Sensoy Bahar, Yesim Tozan, Proscovia Nabunya, Larissa Jennings Mayo-Wilson, Joshua Kiyingi, Joseph Kagaayi, Scarlett Bellamy, Mary M. McKay, Susan S. Witte

**Affiliations:** 1 Brown School, Washington University in St. Louis, Campus Box 1196, One Brookings Drive, St. Louis, MO 63130 USA; 2 College of Global Public Health, New York University, New York City, NY USA; 3 Indiana University School of Public Health, Bloomington, IN USA; 4 International Center for Child Health and Development, Masaka, Uganda; 5 Rakai Health Sciences Program, Rakai, Uganda; 6 Drexel University, Philadelphia, PA USA; 7 Columbia University School of Social Work, New York City, NY USA

**Keywords:** Women engaged in sex work, Economic empowerment, Sub-Saharan Africa, Uganda, HIV/AIDS

## Abstract

**Background:**

Sub-Saharan Africa (SSA) has the highest number of people living with HIV/AIDS, with Nigeria, South Africa, and Uganda accounting for 48% of new infections. A systematic review of the HIV burden among women engaged in sex work (WESW) in 50 low- and middle-income countries found that they had increased odds of HIV infection relative to the general female population. Social structural factors, such as the sex work environment, violence, stigma, cultural issues, and criminalization of sex work are critical in shaping sexually transmitted infection (STI)/HIV risks among WESW and their clients in Uganda. Poverty is the most commonly cited reason for involvement in sex work in SSA. Against this backdrop, this study protocol describes a randomized controlled trial (RCT) that tests the impact of adding economic empowerment to traditional HIV risk reduction (HIVRR) to reduce new incidence of STIs and HIV among WESW in Rakai and the greater Masaka regions in Uganda.

**Methods:**

This three-arm RCT will evaluate the efficacy of adding savings, financial literacy and vocational training/mentorship to traditional HIVRR on reducing new incidence of STI infections among 990 WESW across 33 hotspots. The three arms (*n* = 330 each) are: 1) Control group: only HIVRR versus 2) Treatment group 1: HIVRR plus Savings plus Financial Literacy (HIVRR + S + FL); and 3) Treatment group 2: HIVRR plus S plus FL plus Vocational Skills Training and Mentorship (V) (HIVRR + S + FL + V). Data will be collected at baseline (pre-test), 6, 12, 18 and 24-months post-intervention initiation. This study will use an embedded experimental mixed methods design where qualitative data will be collected post-intervention across all conditions to explore participant experiences.

**Discussion:**

When WESW have access to more capital and/or alternative forms of employment and start earning formal income outside of sex work, they may be better able to improve their skills and employability for professional advancement, thereby reducing their STI/HIV risk. The study findings may advance our understanding of how best to implement gender-specific HIV prevention globally, engaging women across the HIV treatment cascade. Further, results will provide evidence for the intervention’s efficacy to reduce STIs and inform implementation sustainability, including costs and cost-effectiveness.

**Trial registration:**

ClinicalTrials.gov, ID: NCT03583541.

## Background

The highest number of people living with HIV/AIDS (24.7 million) is in sub-Saharan Africa (SSA), with Nigeria, South Africa, and Uganda accounting for 48% of new infections [1]. In Uganda, the HIV prevalence among 15–49 year-olds is 7.2%, with Rakai (9.3%) and Masaka (12%) [2] districts above the national average [1].

A systematic review of the HIV burden among women engaged in sex work (WESW -defined as women who exchange sex for money or goods) in 50 low- and middle-income countries found that WESW had increased odds of HIV infection (OR 13.5, 95% CI 10.0–18.1) relative to the general female population [3]. A study among WESW in Kampala, Uganda, found HIV prevalence to be as high as 37%, with significant presence of other sexually transmitted infections (STIs), including Gonorrhea (13%); Chlamydia (9%); Trichomonas (17%); and bacterial vaginosis (56%) [4]. In more rural regions and HIV “hotspots,” including those targeted by this study, the prevalence of HIV among WESW is as high as 61% [5]. STIs and alcohol use are co-factors for HIV risk globally, but also in Uganda [6, 7], where drinking rates among WESW are as high as 54% [6, 8]. STIs [8] and lifetime IPV [9] rates are significantly higher among WESW compared to the general population. While WESW in Uganda have long been the subject of surveillance studies, this highly vulnerable population has so far not been targeted by innovative and sustainable prevention intervention approaches despite the calls from researchers in the region [10–13].

Social structural factors, such as the sex work environment, violence, stigma, cultural issues [14–18] and criminalization of sex work [19], play a crucial part in shaping STI/HIV infection risks among WESW and their clients in Uganda. Poverty is the most commonly cited reason for involvement in commercial sex work in SSA [20–23]. In Uganda, where poverty and unemployment rates are disproportionately high among women [24], transactional sex is a survival strategy [25, 26]. A growing body of evidence suggests that HIV prevention interventions must address risk factors beyond the individual level to be effective [27, 28]. Gender inequalities in particular have affected women’s social, economic and political opportunities, making them more disadvantaged than their male counterparts [14, 15, 29, 30]. Females engage in high-risk sex for economic survival, and perceive their acts as a strategy to improve their socio-economic well-being [31]. As in other settings, in Uganda WESW are offered at least twice as much money for unprotected sex [32]. The economic advantage of higher risk sex in the face of high HIV prevalence calls for structural interventions offering alternative forms of income for WESW as a public health imperative.

Evidence-based microfinance for enhancing HIV prevention may better address structural factors that hinder traditional prevention efforts for WESW [33, 34]. Microfinance programs constitute one of the fastest growing anti-poverty strategies in developing countries [35]. Microfinance interventions have led to reductions in sexual risk behaviors among poor women and those engaged in sex work [26, 36–40]. Microfinance interventions in Kenya and South Africa have resulted in reduced number of sex partners and higher consistency in condom use [41], improved HIV-related communication, increased voluntary counseling and testing, and decreased unprotected sex [42]. Similar findings were reported from a study in Baltimore, US [38], and India [40]. There are important limitations to a MF approach that focuses specifically on microloans, particularly for poor women who experience intersectional marginalization due to their sex work [43–45].

The proposed study innovates by proposing interventions that use a savings-led approach, which has the benefit of enabling participants to accumulate assets faster and pay for life-cycle events without accumulating debt and an over-reliance on borrowing [45]. Savings-led approaches have demonstrated efficacy in reducing sexual risk behaviors among young women in Uganda [46, 47] and among WESW in Mongolia [48–50]. Savings-led MF approaches for economic empowerment are in line with Uganda’s Government Vision 2040 that calls for investment in financial inclusion for the most vulnerable groups. Thus, such approaches should be a priority for testing among poor vulnerable groups, including WESW, before being taken to scale.

Against this backdrop, the team presents, in detail, the study protocol for a 3-arm randomized controlled trial (RCT) that tests the impact of adding economic empowerment components to traditional HIV risk reduction (HIVRR) to reduce new incidence of STIs and of HIV among WESW in Rakai and the greater Masaka regions in Uganda. The study arms are: 1) a control arm comprising HIVRR sessions provided by community health workers; 2) treatment arm 1 that includes HIVRR, combined with receipt of a matched savings account and financial literacy with integrated behavioral economics principles (HIVRR + S + FL); and 3) treatment arm 2 that includes HIVRR, combined with a matched savings account, plus financial literacy with integrated BE principles, and Vocational Skills Training and Mentorship sessions (V) (HIVRR + S + FL + V). More specifically, the study aims are as follows:

1. **To examine the impact of a financial savings-led microfinance intervention using HIVRR + S + FL and HIVRR + S + FL + V on HIV biological and behavioral outcomes in WESW using a RCT.** The primary outcomes will be women’s: 1) cumulative incidence of biologically-confirmed STIs (Gonorrhea, Trichomonas, Chlamydia); and 2) reported number and proportion of unprotected sexual acts with regular and paying partners. Secondary outcomes will be women’s: 3) rate of new HIV cases; 4) proportion of monthly income from sex and non-sex work; 5) reported number and proportion of preventive behaviors (condom purchasing, HIV testing, partner discussions, and Pre-Exposure Prophylaxis (PrEP) use; and 6) for HIV positive women only, viral load as a marker of ART adherence.
2. **To examine intervention mediation and effect modification.**We will statistically assess whether the primary outcomes are mediated or moderated by participant characteristics; and whether key theory-driven variables and behavioral economics measures mediate or moderate intervention outcomes.
3. **To qualitatively and quantitatively examine implementation in each study condition.**We will investigate participants’ interventional experiences (satisfaction, facilitators, barriers, recommendations); factors influencing participation, sexual decisions, financial behaviors; and perceptions on programmatic sustainability.
4. **Assess the cost and cost-effectiveness of the HIVRR + S + FL and HIVRR + S + FL + V intervention compared with traditional HIVRR.** Using a Markov state-transition model, we will estimate the incremental cost per disability-adjusted life year averted in a hypothetical cohort of female sex workers over lifetime from the health care provider perspective.

### Theoretical framework

The study is guided by social cognitive [51, 52] and asset theories [53, 54].

**Fig. 1:**
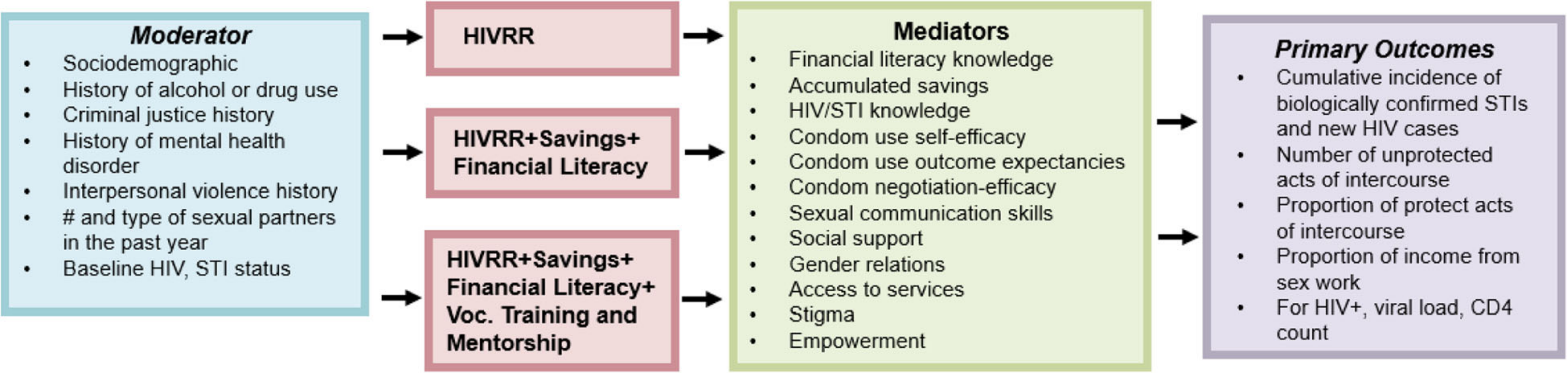
Conceptual Framework

***Social Cognitive Theory*** [51] has guided many HIV prevention studies and includes social cognitive mediators listed below (see Fig. 1). The central tenets of social cognitive theory, including self-efficacy and outcome expectancies, are measured in this study for both paying and intimate partners. Self-efficacy, for example, have been found to affect whether people consider changing their behavior, the degree of effort they invest in changing, and long-term maintenance of behavior change [55]. Self-efficacy with respect to negotiating and using condoms with partners –intimate or paying– has been found to be a strong predictor of condom use [56, 57] and is often found in conjunction with empowerment in sexual relationship decision making [58]. The economic empowerment components for the proposed study have been adapted to integrate self-efficacy with outcome expectancies related to building financial literacy, vocational knowledge, and business development skills. For example, participatory sessions, characterized by lecture, discussion, modeling and role plays, include information on financial literacy skills and emphasize realistic goal-setting and ongoing savings to generalize lessons into daily life.

***Asset theory*** [53, 54] posits that economic assets may yield a range of outcomes, including increased economic stability. These, in turn, may mutually reinforce non-economic assets, including psychological, behavioral, and social assets [53, 54]. For low-income women, assets gained from economic empowerment are rich and complex, and have been operationalized to include economic, health, gender-based and psychological empowerment [59, 60]. For WESW, intersectional stigma and oppression increase the interpersonal and structural barriers to achieve such gains. In the current study, asset theory recognizes that there may be psychological, behavioral and social asset improvements in mediators for the three study arms, e.g. condom negotiation self-efficacy, social support, access to services, as illustrated in Fig. 1. Asset theory has been successfully applied in economic empowerment interventions in Uganda [46, 61, 62], resulting in sexual risk reduction among Ugandan adolescents [46, 63] and HIV risk reduction among WESW in Baltimore, Kenya, South Africa and Mongolia [37, 38, 41].

## Methods/Design

### Study design

**Fig. 2:**
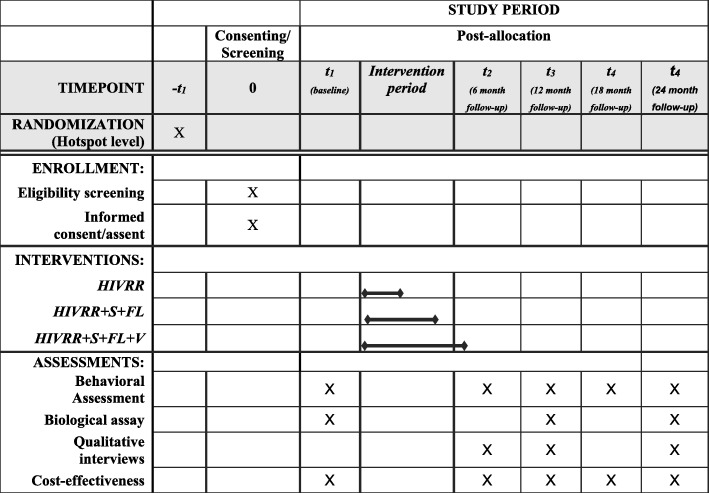
Schedule of enrollment, interventions, and assessments

This is a three-arm RCT that will evaluate the efficacy of adding savings and financial literacy and mentorship to traditional HIV risk reduction on reducing new incidence of STIs among 990 WESW in the Masaka region of Uganda. The three arms are: 1) control group WESW receiving only HIV Risk Reduction (HIVRR) (*n* = 330) versus 2): HIVRR plus Savings (S) plus Financial Literacy (FL) (HIVRR + S + FL) (*n* = 330); or 3) HIVRR plus S plus FL plus Vocational Skills Training and Mentorship (V) (HIVRR + S + FL + V) (*n* = 330). There will be five assessment points: baseline (pre-test), 6, 12, 18 and 24-months post-intervention initiation (see Fig. 2).

This study will use an embedded experimental mixed methods design [64] where qualitative data will be collected post-intervention across all three arms. The qualitative data will explore: a) participants’ experiences with each of the study arms and their specific components, including how women make spending decisions; b) key multi-level factors that may have hindered and facilitated WESW’s participation in each intervention component (HIVRR + S + FL + V); c) savings and risk-taking decisions and behaviors post-intervention (follow-up); d) WESW's perceptions regarding economic costs and rewards, relevance of including salience of positive or negative feedback, relating to preventive sexual behaviors; and e) perceptions on sustainability of each intervention. Data integration will occur at the interpretation and discussion stages for complementarity and expansion [65, 66].

To maximize cultural relevance, feasibility, and adherence to ethical issues [67], the team will form a community collaborative board (CCB) that includes WESW, non-governmental organizations (NGOs), local police, government, training centers, and banks. The CCB will meet quarterly to inform and shape study protocols and to guide study implementation.

### Study setting

The study will be conducted with 990 self-identified WESW recruited from 33 comparable hotspots located in Rakai, the Greater Masaka and Mbarara Regions. In Uganda, the HIV prevalence among 15–49 year olds is 7.2%, with Rakai (9.3%) and Masaka (12%) [2] districts above the national average [1]. Overall HIV prevalence is 12 times higher among WESW compared to the the rest of the adult population, with 37% sero-prevalence among WESW in Kampala [4], and 77% of WESW reporting new STIs in the past year [66]. HIV prevalence among WESW in Rakai and Masaka regions is as high as 61% [5].

### Randomization

After HIV hotspots have been identified, we will use a block randomization approach to allocate each hotspot town to one of three interventions: HIVRR, HIVRR + S + FL or HIVRR + S + FL + V. Specifically, the 33 hotspots will be matched into triplets based on the following characteristics: whether they are predominantly rural or urban, and the estimated number of WESW, so that each triplet member of a triplet is similar. To reduce the potential for contamination, no two hotspots in any triplet will be within the same district. Following triplet matching, towns will then be randomized to one of the three study arms. Each hotspot’s assignment to condition will remain blinded to research staff, with the exception of MPIs and Project Coordinator, until after enrollment (described below) takes place.

### Study population, recruitment, and retention

#### Study population

Our implementation partners, Rakai Health Sciences Program (RHSP) and Reach the Youth (RTY), report that there are roughly 1895 registered women receiving services at hubs within the 33 hotspots targeted for the study. These hubs are located within the 33 hotspots where we will conduct study recruitment. Based on demographic statistics from both institutions, the team expects between 80 and 90% of women to meet study eligibility criteria. The team further expects possible attrition from screening to enrollment of 10–20%. However, we use conservative estimates in anticipation of enrolling at least 990 women (minimum of 55% eligibility) into the study.

#### Recruitment and inclusion criteria

The study will utilize multiple recruitment strategies informed by our pilot studies in Mongolia (led by Witte and Ssewamala) and Uganda (led by Ssewamala). Specifically, we will rely on: 1) recruitment by the International Center for Child Health and Development (ICHAD) in each hotspot – already trained in human subjects protocols and who have worked with vulnerable populations; and 2) asking eligible women to refer other women from the same hotspot who may also be engaged in sex work.

As each grouping of three hotspots is randomized, outreach will be made to the three locations within that triplet. In the initial contact meeting, groups of women will be invited to attend informational sessions. Based on our prior experience in the study region, it is possible that many of the participants will not speak English. Therefore, the information sessions will be conducted in English and Luganda (the local language) depending on a participant’s comfort level and proficiency. Research Assistants (RAs) from ICHAD will provide information on study participation, administer consent and screen women to determine eligibility. The screening interviews for eligibility will be held at private RHSP offices in Kalisizo, ICHAD offices in Masaka, or participating NGO offices, depending on the women’s location in the study region. The screening interview will contain eligibility related items, socio-demographics, and items that will camouflage the eligibility criteria.

*Women will be eligible if they*: 1) are at least 18 years old; 2) report having engaged in vaginal or anal intercourse in the past 90 days in exchange for money, alcohol, or other goods; and 3) report at least one episode of unprotected sexual intercourse in the past 90 days with either a paying, casual, or regular sexual partner.

A participant will be excluded from participation in the study if: 1) she is assessed to have a severe cognitive or psychiatric impairment that would interfere with her ability to provide informed consent or complete study instruments. As in prior studies directed by the PIs, a standardized diagnostic tool will not be used to determine presence of a cognitive or psychiatric disorder. Rather, as part of informed consent, a potential participant is asked to state her understanding regarding three areas covered earlier during the informed consent protocol: (1a) the nature and extent of participation in the study; (1b) the risks involved with participation; and (1c) the potential benefits of participation in the study. If a participant is unable to respond to any of the three items by reiterating the information presented earlier, she will be excluded from the study. During the consent process women will have an opportunity to ask questions and discuss any concerns or confusion with the Project Coordinator who will lead recruitment and consent. Other participant exclusion criteria include; 2) she is unwilling or unable to commit to completing the entire study; and 3) she has been previously randomized to one of the hotspots.

Following recruitment, participants meeting inclusion criteria will be consented to participate and scheduled to complete baseline interview (including bio-testing) within 14 days. Blood and vaginal swab specimens are collected and taken the same day to the reference laboratory at RHSP. Women testing positive for an STI will receive single dose treatment. While not a study requirement, women testing positive for HIV will be referred for medical care, including access to anti-retroviral treatment (ART). Women testing negative for HIV will be referred for pre-exposure prophylaxis (PrEP).

Following baseline in all sites, women will be scheduled for the four sessions of HIVRR to begin within 30 days; and completed within 30 days from the start date. Given the use of cluster randomization, all women from the same hotspot will be assigned to the same study condition. Disclosure of condition will happen at the first intervention session.

#### Retention

Earlier studies in the region by our collaborating partners indicate that young women who attend health-related services demonstrate attendance rates of 94% and women who attend multisession activities have slightly lower attendance rates by ~ 3 percentage points (91%) [5, 46, 47, 62, 67–71]. Moreover, based on our prior work with WESW, once women are meaningfully engaged, they continue to attend intervention sessions. A recent review of WESW interventions in SSA shows that those with multiple components, of high intensity and coverage, yield more desired outcomes compared to those with a limited number of components [72].

We will use retention strategies currently utilized by RHSP [73, 74] and the Suubi+Adherence study [75, 76] to successfully follow participants. During consenting, the team will ask participants to provide future contact information, including phone number(s), and list of the names, addresses, and contact information of three people who will always know how to reach them. Participants will be reminded that in any contact made, the team will not discuss details about them or their study participation. To further reduce potential attrition, we will reimburse women for transport to sessions.

Effective use of these procedures in our previous research studies has resulted in very low attrition rates (9.7% over 5 years) [69, 77–80]. The team will keep drop-out records and test for attrition bias based on baseline data. If such bias is present, the team will limit generalizations accordingly or, where possible, introduce statistical adjustments to address bias. Strategies outlined here follow the protocols used in the *Bridges* and *Suubi-Uganda* studies [47, 81, 82].

### Ethics and informed consent

All study procedures were approved by the Washington University in St. Louis Institutional Review Board (IRB #201811106), Columbia University IRB (IRB #AAAR9804) and the in-country local IRBs in Uganda: Uganda Virus Research Institute (UVRI #GC/127/18/10/690), and the Uganda National Council of Science and Technology (UNCST #SS4828). All potential amendments to the study protocol will be submitted for approval to the above-mentioned IRBs by the MPIs.

All forms used to provide information on the study as well as consent forms have been made available in English and Luganda. A certified translator from the Department of Languages at Makerere University has translated the forms into Luganda and then back translated them to ensure accuracy. One of the Multiple Principal Investigators (MPI; Ssewamala), the in-country Principal Investigator (PI; Kagaayi) as well as the Project Coordinator speak Luganda and will check the forms.

The Project Coordinator and RAs (all fluent in Luganda) will be completing the consenting. Research staff will be trained by the MPIs/designee about the consenting process. Training will be ongoing to ensure that research staff follows the guidelines for consenting. All research staff hired for the project will complete the CITI training.

Written consent from participating women will be obtained by the research staff prior to or at the time of their participation in the study. Consent forms will be available in the local language for all study participants. Women will be assured that their decisions about participation (yes or no) will in no way affect their relationship with any health facility and/or other service facilitators. The consent forms will describe all aspects of the study using literacy appropriate language, including procedures for handling data and explain that confidentiality will be maintained unless concerns about the participant warrant reporting, such as suicidality or homicidality or abuse. The consent will describe the purpose of the study, the participant’s involvement, where the study will be conducted, how much time participation is expected to entail, and the information they will be asked to provide. Sufficient time will be allowed for questions about the consent forms or about the study in general.

In the consent form, it is clearly stated that the participant can withdraw from the study at any time, for any reason, with no explanation, and will not be penalized in any way. It states that a participant may refuse to answer any questions at any time, may review any materials, may request that we erase any of their responses and may make inquiries and address complaints to Executive Secretary, Uganda National Council for Science and Technology, the Human Research Protection Office at Washington University or Columbia University’s Committee for the Protection of Human Subjects, or UVRI. As mentioned earlier, the research team will also inform the participant of any potential risks and benefits of participating in the program. Each participant will receive a copy of the consent form and will be thoroughly briefed regarding the importance of consent being both informed and voluntary. The participant will be excluded from the study if a potential participant is unable to state the following after reviewing the consent form: 1) the nature and extent of participation in the study; 2) the risks involved with participation; and 3) the potential benefits of participation in the study. Participants will also be asked about agreeing for interviews and intervention sessions to be audio recorded solely for the purposes of quality assurance. A separate consent form indicates that such agreement is not a requirement to participate in the study and informs the participant that any portion or the entire recording will be erased upon her request at any time, including during or any time after the session.

In cases where literacy is of concern, the research staff will walk the participant through the consent by reading out the consent letter or will have a literate member of the family or an impartial witness of the participant’s choosing read the consent. Consent letters will be signed (or thumb printed) by the participants, and the research staff obtaining consent. Subjects refusing to consent will be thanked for their time and withdrawn from participation. Original copies of the consent forms will be kept in the locked file cabinets of a locked office in a secure building at ICHAD in Uganda. Copies of the forms will be given to women.

### Intervention conditions

HIV/AIDS information materials available through the Ugandan Ministry of Health will be distributed to all study participants to ensure that we standardize access to basic information across all three study arms. In addition, all WESW, regardless of the study condition will receive HIVRR sessions (see below for details).

#### Control condition (HIVRR)

**Fig. 3:**
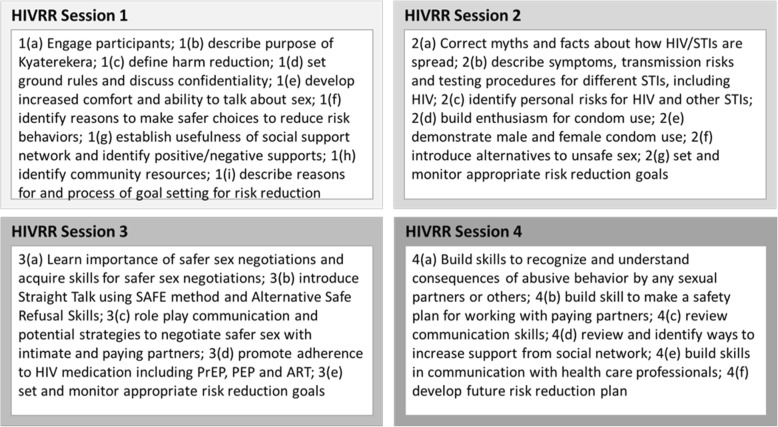
HIVRR sessions overview

Women in the control condition (and in the treatment arms) will receive 4 sessions of HIV Risk Reduction (HIVRR) (see Fig. 3) provided twice per week for 2 weeks of an evidence-based, HIV/STI risk reduction intervention tested in three previous studies by Witte [34, 48, 49]. During session 3, linkage to PrEP and ART/medication adherence skills will also be provided.

The HIVRR session will be delivered by the community health workers who will be trained by the study team.

#### HIVRR+S + FL treatment condition

Women in this arm will receive the HIV/AIDS information, HIVRR sessions (described above) and the financial literacy training (described below). Women in this arm will also save money in their matched savings accounts (described below). The study team will monitor the accounts using the statements received directly from the banks holding the accounts. Participants will receive monthly bank statements indicating their own savings and the associated match (1:1 match rate).

#### HIVRR+S + FL + V treatment condition

Women in this arm will receive the 4 HIVRR sessions (as above). Next, they will receive the Savings (S) session and 7 Financial Literacy (FL) sessions provided twice a week for 3 weeks, followed by 8 Vocational Skills Training and Mentorship sessions (V) sessions supporting transition to vocational, educational training, employment or business development, and receipt of a matched savings account to be used on short-term and/or long term consumption and skills development per participants own discretion/choice.

##### Financial literacy

Adapted for testing with WESW in Undarga, this widely translated evidence-based Financial Education Core Curriculum [83] addresses the importance of savings, banking services, budgeting (including household budget development) and debt management (see Table 1). Undarga adaptation for WESW included shortening and simplifying sessions while retaining core elements; adding weekly check-ins due to safety concerns WESW share related to intervention participation, and safety planning as needed. The team will further adapt sessions in months 1–6 with the CCB to assure language and illustrative examples are culturally and regionally consonant, and to infuse behavioral economics principles consistent with HIV risk reduction. The BE content is focused on encouraging uptake of safe sexual and income-earning practices, including but not limited to delaying small immediate awards (higher pay for unprotected sex) for larger awards long-term (e.g., benefits to sexual health or alternative forms of employment); replacing/exceeding income lost from unprotected sex – economic utility; and considering individual economic costs (such as disease burden, lower productivity, stigma) of losing good sexual health through unsafe sex.

**Table 1:** FL Intervention Content

Session#	Content
1	Banking: Explore Common Perceptions about Banks and share personal banking experiences; Evaluate why a bank is better than a “piggybank”, “under the pillow” or “mattress account”; Introduction to local financial institutions and opening bank accounts; Safety and safety planning
2	Savings and Financial Goal Setting: Defining savings and why people save; Identifying challenges to savings, Setting savings goals related to family and vocation; Personal financial goal settings
3–4	Budgeting and Financial Planning: Examine Money Management and Balancing a Budget; Set Financial Planning Goals; Describe Importance of Budgeting; Staying within budget and cut spending.
5	Debt Management: Borrowing Money: Things You Need to Know; Managing Loans and Debt; Costs of Borrowing; Delinquency: What Is It and How Does It Happen? The Dangers of Over-Indebtedness and Default
6	Emergency Funds: Planning for Emergencies, Maintaining an Emergency Fund and Adjusting Savings Goals; Planning for the Future.
7	Behavioral Economics: Delay Discounting; Economic Utility; Information Salience; Loss Aversion

##### Vocational skills training and mentorship sessions (V)

This includes three transition sessions to a specific vocation/goal augmented with five additional vocational mentorship (hands-on) sessions from a “role model” peer that our collaborating field partners (RTY) will help to identify. The first 3 sessions focus on identifying options for vocational, educational, employment or business development training. The WESW will be matched with the role model from the same vocation that they express interest in for the following five sessions. The vocational skills mentorship is intended to be supportive of the women as they transition into a specific formal vocational training, engage with formal training/education, and eventually launch into formal employment or business development.

##### Matched savings individual development account (IDA)

IDA is a savings account held at a local bank whereby deposits made by the woman are matched by the intervention to encourage savings and investment in skills and asset development. The accounts introduce women to financial management skills, introduce them to formal financial institutions, and by matching their deposits, incentivize women to save small amounts. Each woman assigned to either treatment group will receive an IDA held in her own name. We have partnered with nationally registered banks operating in the study area: Centenary Rural Development Bank, Development Finance Company of Uganda Bank (DFCU), Diamond Trust Bank (DTB) and/or Stanbic Bank to host these matched savings accounts. Women will be allowed and indeed encouraged to contribute up to 80% of the total incentives received from their participation in the study. This would include money received from: the 4 HIVRR sessions + 7 FL sessions + 8 V sessions. The savings will be matched during the month they receive the incentives. Depending on the study condition, the maximum amount of a woman’s contribution to be matched (the match cap) will be an equivalent USD $15 per session of HIVRR + S + FL; or HIVRR+ S + FL + V. To illustrate, a woman in the treatment 1 condition could potentially earn an equivalent of $15 × 4 (HIVRR sessions) + $15 × 1 (savings transition) + $15 × 7 (financial literacy sessions). That would give a woman an equivalent of $180 over the 12 sessions with which the woman would have had contact with the intervention team. Women who save the maximum allowable amount ($15 × 12 sessions/direct contacts would potentially accumulate $180). This would then be matched by the intervention at a rate of 1:1, potentially giving the woman a total equivalent of $360. At the current exchange rate, this is an equivalent of 1,260,000 Ugandan Shillings (UGX). Similarly, a woman in the treatment 2 condition could potentially accumulate $300 derived from attending 4 HIVRR sessions, 1 savings session, 7 financial literacy sessions, and 8 vocational skills training and mentorship sessions (20 sessions in totalx$15 = $300). This amount would then be matched by the intervention, potentially giving the woman a total of $600 (UGX 2,100,000).

Each month during the intervention period an account statement will be generated for each woman to note her accumulated savings (own savings plus the match). Monthly statements act as “morale boosters”. Unique to this study is our *innovative* spending model, which empowers women with agency to make informed financial decisions. During the intervention, women will have direct access to both their personal savings deposited in the accounts and the match provided by the study. This is different from our prior studies that required the participants’ own savings and the match to be kept in separate accounts and to get approval by the research team to access the match [46, 47, 63, 78, 84–89]. This added unconditional component provides women with a safety net to address short-term consumption needs and financial emergencies if they arise.

With a total of 15 FL + V sessions tailored specifically to the needs of WESW in Uganda, we expect women will be equipped with the knowledge to make well-informed investment decisions, but also feel supported in case of immediate consumption needs. The research team will *monitor, but not restrict* how women spend their match via assessment questions and qualitative interview questions. Also, the study team will have access to and review participants’ bank statements to ascertain deposit and withdrawal frequency.

### Procedures to maximize internal and external validity

Additional measures will be taken to monitor the following threats that may compromise internal validity. The control and treatment interventions are provided in distinctly different hotspots, per the randomization procedure, reducing the threat of contamination. Since these towns depend on the same economic activities, when migrations happen due to business seasonality, women tend to move to the capital city, Kampala, not within the targeted hotspots. However, in addition to monitoring all participants’ attendance reports of any exposure to session content (through assessment survey, process measure and anecdotally among staff) inconsistent with a participant’s random assignment will also be assessed and monitored. Staff has been trained on the experimental nature of the interventions and the importance of not introducing S and FL + V content to the HIVRR participants. The research team will implement rigorous quality assurance process throughout the study. If quality assurance (QA) monitors discover contamination, they will identify how facilitators responded and address with staff; follow-up assessments will include a brief survey containing six items asking if they discussed any knowledge, skills or information that they learned in the sessions with other participants, and if so, what topics.

### Data collection

#### Process measures and quality assurance

The research team has adapted QA procedures used successfully in the team’s ongoing HIV/STI intervention studies and has provided comprehensive training prior to start up following detailed protocols for all procedures. Process measures will be used to monitor the fidelity and quality control of intervention implementation and will capture: 1) *attendance/dosage* using a participant attendance form to monitor session attendance; 2) *adherence and contamination* using a Session Adherence Checklist consisting of number, duration, and sequence of session activities and perceived quality of delivery, including potential contamination. Both will be used at the end of each session and facilitated by the study team; and 3) *participant satisfaction* using a questionnaire to assess attitudes towards and satisfaction with the HIVRR curriculum and each treatment component (savings, financial literacy, behavioral economics, and mentoring). All session data will be reviewed on an ongoing basis by the team led by the MPIs.

#### Outcome measures

Completed at baseline, 6, 12, 18 and 24 months post-intervention initiation, assessments will include sociodemographic data, outcome measures, and putative moderators and mediators specified by our theoretical framework (see Table 2) [87, 123]. Self-reported sexual risk outcome questions are used in the MPIs current HIV prevention trials and ask specifically about the number and type of sexual acts in the past 90 days, as well as of protected sexual acts in the past 90 days with various partner types. They will be interviewer-administered, utilize a computer-assisted data entry system employed by the team in current clinical trials; and will be conducted in a private space at satellite field offices, typically in 60 min. All female interviewers will minimize discomfort about potentially sensitive information. Participants will be compensated for each assessment. Marlowe-Crowne Social Desirability Scale [98, 99] will be administered at each assessment point to assess whether or not respondents are concerned with social approval.

**Table 2:** Study Measures

Assessment Variables	Measurement	Reliability	Time point
Moderators			
Age, income, education, marital status, history of sex work	Sociodemographic Questionnaire		B,6,12,18,24
Alcohol use, substance use, criminal justice history	Modified NIDA Risk Behavior Assessment (RBA) [90] Alcoholic Use Disorder Identification Test (AUDIT) [91, 92]	.66–.83	B,6,12,18,24
Mental health status	PTSD Checklist [93] Brief Symptoms Inventory (BSI) [94]	.97	B,6,12,18,24
Partner violence history (paying and intimate)	Revised Conflict Tactics scale [95]	.80	B,6,12,18,24
# and type of sexual partners (past year)	Modified NIDA Risk Behavior Assessment (RBA) [90]	.66–.83	B,6,12,18,24
Perceived stigma	Sex Worker Stigma Index [96] HIV Stigma Scale [97]	.87–.88 .50–70	B,6,12,18,24
Social desirability	Marlowe-Crowne Social Desirability Scale (Short form C) [98, 99]	.51	B,6,12,18,24
Mediators			
HIV/STI knowledge	Brief HIV Knowledge Questionnaire [100] Questions used in NOVA and Suubi studies [33, 34, 101–103]	.80	B,6,12,18,24
Condom Use Self-efficacy	Condom Use Self-Efficacy Scale [57]	.94	B,6,12,18,24
Condom Negotiation Self-efficacy	Questions adapted from Couples Communication Scale [104]	.80	B,6,12,18,24
Sex life/behavior	Questions adapted from Levy et al. [105], Paikoff et al. [106], and Rotheram-Borus et al. [107] Scale adapted from the CDC Violence Against Children Survey [108] Questions adapted from NOVA [33, 34]	.80	B,6,12,18,24
Social support	Multi-Dimensional Scale of Perceived Social Support [109] Family relations/cohesion scale [47, 62, 80] Questions adapted from Suubi and Bridges studies [68, 75, 76, 80, 87]	.84	B,6,12,18,24
Gender-based violence	Domestic violence attitudes questions adapted from NOVA and COMPASS studies [33, 34, 110] Intimate partner violence items adapted from Revised Conflict Tactics scale [95], Economic abuse items adapted from Scale of Economic Abuse [111]	.80 .79–.95 .93	B,6,12,18,24
Attitudes towards gender roles; decision-making; communication	Gender Relations Scale [112]		B,6,12,18,24
Access to medical care	Survey questions adapted from Kalichman et al. [113] and Cunningham et al. [114]	0.74	B,6,12,18,24
Financial self-efficacy	Adapted from Financial self-efficacy subscale of the Domestic Violence-Related Financial Issues scale [115]	.75–.86	B,6,12,18,24
Savings Deposits	Bank Statements		B,6,12,18,24
Behavioral economics measures	Questions adapted from Behavioral Biases [116, 117] and Delay Discounting Task measures [118–120]		
Vocational, educational, or business development sessions attended	Session attendance		6,12,18,24
Outcomes			
STI (gonorrhea, Trichomonas, chlamydia) HIV status	Biological Assay		B,12,24
STI testing and treatment	Chart Review		24
Sexual Risk: #, % of unprotected sexual acts by partner type; #, % protective practices; e.g. PrEP uptake, ART adherence, condom use	Modified NIDA Risk Behavior Assessment (RBA) [90] Questions adapted from Knowledge, attitude and use intention of HIV prevention methods survey [121] Questions adopted from NOVA, Undarga, and Suubi [33, 34, 48, 75, 76]	.66–.83	B,6,12,18,24
Cost of staff time, supplies, overhead for HIVRR and for SMF	Project records; Admin. review		Ongoing
Viral load, CD4 count (HIV positive women only) [122]			B,12, 24

#### Biological assay

Collection, counseling, notification, referral for treatment, follow up and monitoring procedures for biological testing for HIV, Gonorrhea, Trichomonas, and Chlamydia – all used at RHSP and in current studies by the MPIs – will be completed at baseline, 12 and 24 months post-intervention initiation. RHSP staff will also conduct chart review at 24 months for all participants to ensure that we identify any STI testing and treatment falling outside the study protocol.

#### Semi-structured interviews

Semi-structured in-depth interviews will be conducted at the end of the intervention, and at 6, 12 and 24-month follow-up for each study group. The first interview will focus on: 1) participants’ experiences with the respective intervention and its specific components (i.e., HIVRR, savings, financial literacy, and mentoring) and 2) key multi-level (individual, economic, family, contextual, and programmatic) influences that affected their participation. The follow-up interviews will unpack the longer-term impact, including key multi-level factors affecting participants’ savings and risk-taking decisions and sexual behaviors post-intervention. The follow-up interviews will also inquire into participants’ perceptions of economic costs and rewards of preventive sexual behaviors and perceptions on program sustainability. A purposive criterion sampling strategy [124] will be used: Participants in the highest, mid (average) and lowest quartiles of number of attended sessions in each condition will be identified, and 30 participants (10 from each quartile) from *each* study condition will be randomly selected (*n* = 90; this sample size will be sufficient for theoretical saturation) [125–127]. This sampling method will ensure that participants with varying experiences are represented. This will allow us to identify common patterns and variations in participants’ experiences. Interviews will be conducted in English or Luganda based on participants’ preference. Questions will be translated (English to Luganda) and back-translated by two proficient team members. Each interview will last about 60 min and will be audio-taped. At the end of the intervention, all facilitators (about 9) will be interviewed to gain a deeper understanding of implementation patterns and processes, including their perceptions on sustainability.

### Data analysis

#### Statistical analysis of intervention efficacy

We will employ a logistic regression approach to evaluate Aim 1. Specifically, we will examine whether the presence of a biologically confirmed, incident STI post-randomization is associated with random intervention assignment. Our primary interest is to determine whether or not STI incidence differs for those randomized to: 1) HIVRR  +S + FL or 2) to HIVRR+ S + FL + V compared to HIVRR alone over the 24 month follow-up period. Model estimates will be translated to estimates of incidence risk ratios or odds ratios and 95% confidence intervals (CI) and *p*-values will be calculated for inference. A logistic modeling strategy will also be implemented similarly for HIV incidence outcomes. Because behavioral outcomes of interest change over time and will be measured at multiple time-points, we will employ a similar logistic regression strategy, properly accounting for the repeated measures over time, via a generalized estimating equations (GEE) approach [128]. Specifically, when evaluating self-reported proportion of unprotected sexual acts with both regular and paying partners a logistic GEE will be employed specifying a logit link. Again, we will be interested in estimating rate ratios or odds ratios comparing: 1) HIVRR + S + FL to HIVRR alone or 2) HIVRR + S + FL + V to HIVRR alone. A Poisson GEE modeling strategy (e.g. GEE with specified log link) will be used to evaluate remaining secondary outcomes: self-reported number of unprotected sexual acts with both regular and paying partners. In summary, all models will assess intervention differences (HIVRR + S + FL or HIVRR+ S + FL + V versus HIVRR alone) in outcomes (STI incidence, unprotected sexual acts) longitudinally using a logistic regression strategy or the flexible GEE extension for repeated measures using an over-arching intent-to-treat approach.

#### Power analysis

Power and sample size considerations for this proposal prioritized the primary aim (Aim 1.1 and 1.2). Specifically, the study is powered to examine differences in STI incidence over 24 months post-randomization across interventions (HIVRR + S + FL or HIVRR + S + FL + V vs. HIVRR alone – 2 primary comparisons). Baseline prevalence estimates for this region and population are 77, and 37% for STI and HIV respectively. We have assumed a range of plausible incidence measures that are a fraction of the prevalence estimates in order to estimate the sample size required for this study. Any women who test positive at baseline for an STI will be treated and therefore assumed STI free at randomization. Further, any subsequent positive STI results are considered incident cases. We determined that a sample of 330 WESW per study arm in a cluster-randomized design would sufficiently detect a hypothesized odds ratio of 0.696–0.765 (where the STI incidence in the HIVRR alone ranges from 0.2–0.4 and the STI incidence in the HIVRR + S + FL and HIVRR + S + FL + V groups ranges from 0.15–0.35), adjusting for up to 20% attrition over follow-up with 80% power. This estimate assumes (conservatively) both an intra-class coefficient equal to 0.01 and a Bonferroni correction of the 2-sided Type 1 error for the 2 primary comparisons. We have identified 33 total clusters/towns (11 per intervention) or ~ 30 WESW per cluster, per intervention, so randomizing 330 women in equal proportions to each intervention, in a cluster-randomized design, to HIVRR + S + FL to HIVRR + S + FL + V to HIVRR alone, should provide sufficient power to test the 2 primary hypotheses. Sample size estimates were constructed assuming a cluster-randomized design using the algorithms outlined in Donner and Klar (2000) [129].

#### Statistical analysis of mediation and effect modification

We will explore whether key variables moderate the intervention’s effect on primary outcomes. We will expand the modeling framework proposed for Aim 1 to include ‘moderator X intervention’ interaction terms along with the corresponding main effects. We will use contrasts to assess the magnitude and significance of each moderator on intervention effects through these interaction terms. SAS will be used to fit and evaluate potential moderators of any observed intervention effects. Similarly, we will examine whether key theory-driven variables mediate the intervention’s effects on the primary outcomes, again we expand the modeling framework from Aim 1 to examine: a) whether the HIVRR + S + FL or HIVRR + S + FL + V improves the primary outcome compared to HIVRR alone (Aim 1); b) whether the intervention improves each mediator; and c) whether improvements in the mediator over time are associated with improvements in the primary outcome over time. This general approach to evaluating potential mediators of any observed intervention effects is easily implemented in the logistic and Poisson GEE modeling framework in SAS as recently illustrated in another HIV risk modification trial [130].

We hypothesize that using local agencies to provide HIV prevention, matched savings accounts to invest in financial assets (including vocational training and business development), and financial education with integrated behavioral economics principles and mentorship will additively lead to significant and sustained reductions in study outcomes compared to HIVRR alone. Specifically, we hypothesize that the post-intervention odds of having STIs and HIV risk behaviors will be lower in the two treatment arms vs. the control arm, and lowest in the HIVRR + S + FL + V arm.

#### Handling missing data

Our RAs and Project Coordinator will be trained to check assessments for missing items before leaving the participant. If any missing items are identified, participants will be asked to answer those items–unless they chose not to answer those particular items. The Project Coordinator will also review assessments within 48 h of completion. If missing items are identified in the baseline assessment (time point 1), research assistants will follow up with the participants before the first session of the intervention. If missing items are identified at the 6-month assessment (time point 2), 12-month assessment (post-intervention, time point 3) or during 18-month follow-up (time point 4), research assistants will follow up with the participants within one week of the original date of the assessment. If there are still missing data, we will address incomplete data with multiple imputation (MI) because MI makes the relatively mild assumption that incomplete data arise from a conditionally missing-at-random (MAR) mechanism. Auxiliary variables will be included to help meet the MAR assumption and sensitivity analyses will be conducted with weighted MI 177 to assess the robustness of the MAR assumption. SAS and Mplus will be used to perform the proposed analyses.

#### Qualitative data analysis

Interviews will be transcribed and uploaded to QSR NVivo11 analytic software. Analytic induction techniques [131] will be used for coding. Initially,10 interview transcripts randomly selected across the three study groups will be read multiple times and independently coded by the team using sensitizing concepts and identifying emergent themes (open coding) [132]. Broader themes will be broken down into smaller, more specific units until no further subcategory is necessary. Potential themes and subthemes include, barriers as well as facilitators at the individual-level (e.g., motivation, readiness to change, time constraints); economic-level (e.g., savings, sex and non-sex work earnings, condom purchasing, and related BE perspectives), family-level (e.g., competing demands, support); program-level (e.g., content relevance; interaction with other program participants, site-specific concerns); and macro-level (e.g., criminalization of sex work, cultural norms, stigma). In addition to implementation and sustainability, these findings will also shed light into potential mediators and moderators. For facilitator interviews, potential themes/ subthemes include facilitator-level (competency, motivation, training, supervisory support), participant-level (e.g., readiness for change, commitment), site/agency-level (readiness, buy-in, resources), macro-level (e.g., bank systems, cultural norms, stigma). Analytic memos will be written to further develop categories, themes, and subthemes, and to integrate the ideas that emerge from the data [132, 133]. Codes and the inclusion/exclusion criteria for assigning codes [134] will be discussed as a team to create the final codebook in NVivo. Each transcript will then be independently coded by two investigators using the codebook. Inter-coder reliability will be established. A level of agreement ranging from 66 to 97% based on level of coding indicates good reliability [124]. Disagreements will be resolved through team discussions. The secondary analysis will compare/ contrast themes and categories within and across the three groups to identify similarities, differences, and relationships among findings. Member checking, peer debriefing, and audit trail will be used to ensure rigor [127].

#### Analysis of cost and cost-effectiveness

To assess cost-effectiveness of the intervention, we will develop a state-transition Markov model to simulate the risk of STI and HIV acquisition in a hypothetical cohort of 1000 WESW and estimate the clinical benefits (STI and HIV infections and Disability-adjusted life years) and costs over their lifetime, comparing HIVRR-alone to HIVRR + S + FL + V and HIVRR + S + FL, from the health care provider perspective. The efficacy and costs of the interventions for reducing STI/HIV incidence among WESW will be informed by the trial.

Using a micro-costing approach, we will prospectively measure the actual use of resources associated with interventions and service provision in all three study arms. Costs will be drawn from project administrative records and routine program monitoring and evaluation data and collected from every organizational level that will be involved with interventions and service provision throughout the intervention period. There are mainly four main categories of costs: 1) personnel costs will be calculated for staff administering and overseeing the interventions based on time spent and salaries; 2) consumable costs include costs of all consumable items and equipment that have no resale value after one year; 3) overheads include utilities (e.g. water, electricity, communication), rent, and recurrent maintenance costs of facilities and equipment; and 4) capital costs include equipment (e.g. laptops, printers, photocopiers) with an expected useful life of more than one year and will be annualized using a standard discount rate of 3%.

We will add a start-up period, which will include activities conducted before the beginning of the RCT, such as planning and recruitment and training of staff. All these costs will be added to measure the overall resource use and the total cost of intervention and service provision in the three study arms. Distinguishing between start-up costs and implementation costs is important to estimate the cost of scaling up an intervention. We will also distinguish between research costs and routine monitoring and evaluation costs. All research costs will be excluded; however, routine monitoring and evaluation costs will be included as it is expected that these costs will also be incurred in replication and scale up. All costs will be adjusted for inflation to facilitate comparison over time. Other clinical, epidemiological and cost input variables will be derived from the trial cohort or will be obtained from the published studies.

Specifically, we will model the risk of STI and HIV acquisition in a cohort of 1000 WESW by estimating the transition probabilities between five mutually exclusive health states in 12-month cycles over their lifetime: 1) healthy: No STI or HIV infection; 2) STI infection with no concurrent HIV infection; 3) HIV infection with no concurrent STI infection; 4) STI and HIV co-infection; and 5) death. We will track HIV disease progression at the individual patient level through changes in CD4 counts by including a tracker variable in the Markov model. The population characteristics of WESW (e.g. age, initial prevalence of STI and HIV infection, initial CD4 cell count) will be determined according to the baseline data collected on the trial cohort.

WESW will enter the model in one of the first four health states based on HIV and STI infection status. After entering the model, each WESW may remain in the same health state at the end of a cycle, or transition from one health state to another when an HIV or STI infection is acquired, when an STI infection is cleared, and when a death takes place in the cohort. The transition probabilities between the first four health states will be informed by the trial data and calculated based on the incidence risk ratios reported by the trail. In the analysis, we will assume that WESW would have access to appropriate treatment for HIV infection and that STI infections would be treated effectively following detection. While the risk of death due to HIV infection according to CD4 cell count will be derived from the published literature, we will use the life table for Ugandan women for age-specific background death rates. We further will assume that WESW would retire from sex work at age 55 in consideration of the family roles WESW take on as grandparents making them shy away from sex work given the socio-cultural context. We will therefore track WESW individually in the model to assign the same risk of infection with STI and HIV with the general female population in Uganda once they reach retirement age. In the analysis, we will assume that interventions would be offered only once to the cohort of 1000 WESW at the beginning. We will conservatively explore at least two scenarios in which we will assume: 1) no effect after intervention discontinuation; and 2) fully sustained effect for another 12 months and then zero. As described in detail above, intervention costs will be prospectively measured alongside the trial using a micro-costing approach, and these costs would be incurred only once at the beginning of the model. Because of the assumed health care provider perspective, we will consider only the direct medical costs of treating WESW for STI and HIV infections, which will be based on the published literature in the study setting. Since the model will track the cohort of WESW over lifetime, these treatment costs will be calculated over a lifetime time horizon.

Following the standard cost-effectiveness guidelines, future costs and health benefits will be discounted at annual rate of 3%. Strategies will be compared by calculating the incremental cost-effectiveness ratios (ICERs), defined as the additional cost of a specific strategy divided by its additional clinical benefit, compared with the next least expensive strategy. To assess the uncertainty in key variables and the robustness of the results to model assumptions, we will perform a probabilistic sensitivity analysis using a Monte Carlo simulation technique with 10,0000 iterations and the probability distributions based on data from the trial and the published literature [135, 136]: The team will also compare the cost-effectiveness of HIVRR + S + FL and HIVRR + S + FL + V to other interventions targeted at WESW in developing country settings [137–139].

#### Data integration

The qualitative and quantitative data analyses will be done separately. Findings will be integrated at the interpretation and discussion stages [63] Conclusions and inferences will be synthesized for a more contextualized and thorough understanding of the impact of the additive components of the treatment interventions. The mixed methods design will serve two purposes: 1) *complementarity* [64, 65] and 2) *expansion* [64, 65]. Qualitative findings will be connected to quantitative findings where the former will provide explanations and context for findings produced by the latter. More specifically, the qualitative data will potentially provide further explanation and context to the impact of the study [results in primary outcomes (e.g., risk taking) and mediators (e.g., social support, gender roles)] over time with questions focused on multi-level factors impacting decision-making and behavior. Moreover, the qualitative findings will complement and expand on our understanding of attendance and participant satisfaction (also measured quantitatively) for each study group.

#### Potential challenges and alternative strategies

While not anticipating major threats to study implementation, the team recognizes potential concerns and has instituted a stringent retention plan for session attendance. The team expects to achieve enrollment goals and high retention (up to 90% at 24 m post intervention, based on retention of registered clients recorded by RHSP staff and in our ongoing regional studies). Should recruitment, enrollment, or retention deviate from anticipated rates, the team will schedule conference calls to enact solutions. A limitation of this intervention may be that it only appeals to poorer WESW, who are at higher STI risk, for whom income from local vocations or small businesses are competitive. Reduced stigma and other asset gains, however, may yield broader appeal. While lack of condition on spending may lead some women to not spend on needed skills development (at least in the short-run), 15 sessions of FL + V courses tailored specifically to WESW, should equip women with knowledge to make well-informed investment decisions.

### Data safety and monitoring

#### Data safety and monitoring plan (DSMP)

The MPIs will assume primary responsibility for: 1) implementing the DSMP; 2) ensuring that all research staff is trained and comply with human subjects protection requirements; 3) regularly monitoring research activities, data management, and participant experiences during and after interviews; and 4) complying with the reporting requirements of the Washington University and Columbia University IRBs, UVRI IRB and Uganda National Council for Science and Technology (UNCST). The investigators will keep a chronological log of adverse events, which will be summarized and included in the annual data and progress report. Data on adverse events, breaking of confidentiality, and psychological distress will be systematically collected during the study and reported by the MPIs to the relevant IRBs. Data Manager at the ICHAD field office in Uganda will be responsible for monitoring data entry and storage and overseeing Ras responsible for handling data at the Uganda field office. He will submit weekly data reports to the MPIs, and immediately report any data misconduct to the Project Coordinator and the MPIs.

##### Plan for data entry

After each wave of data collection is complete, any hard copies of completed questionnaires will be filed and stored in the ICHAD Uganda field office under the supervision of the Project Coordinator and Data Manager. Electronic data, not containing identifying information, will be stored in a password protected system, to which only the two MPIs, the co-investigators, the statistical consultant, the Data Manager, plus the individuals entering data will have access.

##### Plan for disposition of identifiers at the end of the study

Identifiers for the participants will be disposed of not more than three years after study completion. To protect the participants’ confidentiality, identifiers will only be accessible by the two MPIs, the in-country PI, Project Coordinator and Data Manager, and will be kept separate from other documents containing participants’ responses.

All research staff involved will have completed the CITI Human Subjects training and Good Clinical Practice training courses through Washington University. The Washington University training includes compliance with the Health Insurance Portability and Accountability Act of 1996 (HIPAA). All field staff, quality assurance, and management staff must complete an intensive, day-long, structured training program on detecting, addressing, and reporting adverse events before they are allowed to participate in any research. This training which will be conducted by the MPIs and will also cover how to handle challenging situations, including how to respond to distressed participants and participants who are experiencing life threatening situations. The MPIs will also provide an annual “booster” session for all research staff interacting with participants and handling participant data which will review human subjects protection principles and study-specific procedures, followed by discussion of adverse events for the study over the past year, and role-play exercises.

##### Data confidentiality

Oversight of data management, including data collection and storage, security, tracking, data analysis software and hardware, and QA will be the responsibility of ICHAD staff on site, and the MPIs. Each participant will be assigned a unique study identification number. All paper and electronic files with data will list only the study identification number; no personal identifiers will be included in screening or assessment data. Only the MPIs and the Project Coordinator will have access to the master list linking participants’ identities to their study identification numbers. All hardcopy items with identifying information (e.g., consent forms, tracking information) will be kept in a locked file cabinet in the ICHAD field office. All software files with identifying information will be protected by password and a firewall; backup copies of files will be encrypted and password protected before being archived in a locked file cabinet in the ICHAD office.

Data management activities and procedures will utilize the electronic data management systems at ICHAD to enhance the efficiency, security, and integrity of study data (including quality assurance data), which includes: 1) secure and confidential World Wide Web- (WWW-) based: (a) scheduling information for RAs, (b) automatically generated summary reports for the Project Coordinator to tailor recruitment efforts to maximize efficiency, (c) uploading of digitally-recorded and encrypted interviews for QA protocols, and (d) collection, monitoring, and summary reporting of data relevant to day-to-day operations of the study (e.g., petty cash disbursements); and 2) custom-programmed data entry software to ensure consistency, integrity, and security/confidentiality of the transfer of data recorded by interviewers into a computer database.

The investigative team will also use the internet to transfer and securely store any digital audio recordings used for quality assurance purposes. Digital recordings are transferred and stored on a dedicated FTP server maintained by and accessible only to ICHAD staff. Centrally storing all recordings on a password- and firewall-protected computer enhances security (e.g., all attempted accesses, whether successful or unsuccessful, are automatically logged and reviewed weekly by a dedicated ICHAD computer support staff member) and integrity (e.g., automatic backup of recordings using 128-bit DES encryption onto optical media).

When the study is completed, all digital files with identifying information, including audio recordings, will be destroyed by using software that meets DoD 5220.22-M specifications for deleting files on magnetic media, overwriting files stored on the flash memory used by digital recorders, and physically shredded for files stored on CD/DVD media.

#### Data safety and monitoring board (DSMB)

An independently constituted DSMB will monitor this study, as per recommendations of MPIs. The membership, and reporting requirements will be determined by the MPIs. During the trial, the DSMB will have oversight of the safe conduct of the study. The DSMB will receive a data report from the study team on an established reporting format and schedule. The report will include the major variables necessary for monitoring safety and quality of data collection and integrity of the study, including subject enrollment and retention. All the data reported to DSMB will be de-identified data to protect participants’ privacy and confidentiality. Specific items that will be reviewed by the DSMB include:

1. Interim/cumulative data for evidence of study-related adverse events;
2. Data quality, completeness, and timeliness
3. Adequacy of compliance with goals for recruitment and retention, including those related to the participation of women and minorities
4. Adherence to the protocol
5. Factors that might affect the study outcome or compromise the confidentiality of the trial data (such as protocol violations, unmasking, etc.); and,
6. Interim analysis

### Adverse events

The MPIs are responsible for the clinical management of participants and accurate written documentation, investigation, and follow-up of all possible study-related adverse and serious adverse events (AEs and SAEs). If the SAE/AE is observed by an RA, he/she will contact the Project Coordinator immediately (no later than 12 business hours). The Project Coordinator will then contact the in-country PI as well as the MPIs (Ssewamala and Witte) by email or by phone within 12 business hours of learning about the SAE/AE. The Project Coordinator and the in-country PI will fill out the official paperwork and share with the MPIs. The report will also be filed in study records. All documentation for the events listed above will be made in writing to the IRBs by the MPIs. The MPIs will be responsible for filing the paperwork/report related to the reporting of SAE/AE.

Documentation will include identifying information for the research protocol (e.g., the investigator’s name, project title, the grant/contract number), the date the event occurred and the date the study team became aware of the event, a detailed description of the event and impact on the participants, a detailed description of measures taken, confirmation that the appropriate monitoring entities and regulatory bodies have been notified as needed, and a description of any changes to the protocol or other corrective actions that have been taken or will be undertaken in response to the event.

All data collectors and facilitators will receive extensive training on how to identify verbal and non-verbal signs that may indicate psychological distress and adverse events. They will also be trained on how to support distressed participants and to offer referrals to local clinics/ hospitals if necessary. The in-country teams at RHSP and ICHAD are knowledgeable of resources available to participants in the study region. If the need arises, RAs will make appropriate referrals for basic and enhanced services. In addition, we have trained and certified counsellors and social workers on our research team to provide onsite counseling and comfort in case of psychological distress. If preliminary analyses reveal harm the results will be thoroughly reviewed by the MPIs and CCB whether stopping is warranted. However, no negative results have been identified in prior studies using the HIVRR + S + FL + V intervention.

### Criteria for stopping the study or suspending enrollment or procedures

This study will be stopped prior to its completion if:

1. The intervention is associated with adverse effects that call into question the safety of the intervention;
2. Difficulty in study recruitment or retention will significantly impact the ability to evaluate the study endpoints;
3. Any new information becomes available during the trial that necessitates stopping the trial; or
4. Other situations occur that might warrant stopping the trial.

The local IRB as well as the overseeing IRBs (Washington University, Columbia University, and UVRI) and CCB will review the reported incident and recommend a course of action, including steps necessary to be taken in order to resume research activities. Once these steps are taken by the research team and reported back to the IRB, they will make the decision on whether research activities can resume.

### Criteria for removing a participant from the study

In addition to cases where the participant may choose to withdraw from the study, there may be instances where the study team may decide to remove a participant from the study. If RAs encounter any of these instances, they should immediately contact the Project Coordinator (within 24 business hours). The Project Coordinator will then contact the in-country PI and the MPIs of the project within 24 business hours via email or phone. These cases will be discussed case by case and determination about removal of the participant will be made accordingly. The reason for termination will be documented and all related paperwork will be filed in study records. The participating woman will be considered for removal from the study if she:

1. Expresses intention or attempts to harm herself or others, such as suicidality or homicidality; and
2. When it is determined that the participant requires prolonged psychiatric or medical care

## Discussion

WESW in SSA have been identified as a high-risk group for the spread of HIV/AIDS, with those in poor areas and “HIV hotspots” being especially vulnerable. Research has shown that the primary reason poor women engage in commercial sex work is financial instability. Given these challenges, women living in poverty require support over and above HIV prevention education. Further, while long the subject of surveillance studies, this highly vulnerable population has so far not been targeted by innovative and sustainable prevention intervention approaches to reduce risk and assure access to care and treatment, all of which contributes to eradicating HIV [10–13].

There is substantial evidence, including recent public policy, for sustainability of the proposed treatment conditions—if proven effective. First, the policy environment in Uganda supports gender responsive development initiatives. Citing women’s inadequate access to and control of assets, the Government of Uganda (GOU) has committed to “reduce gender inequalities so that all women and men, girls and boys, are able to move out of poverty and achieve improved and sustainable livelihoods.” [140] Similarly, Uganda’s National Development Plan II pairs women’s gender equality and empowerment with “accelerated socioeconomic transformation.” Aligning national goals with the UN 2030 Sustainable Development Goals, Ugandan policy makers stated their aim to give women equal rights to economic resources [140]. Further within the NDP II, the Social Development Sector is charged with increasing the percentage of women accessing economic empowerment initiatives to 30% by 2020 [141].

In coordination with Ugandan stakeholders, USAID has also made recommendations to the GOU in the context of the Country Development cooperation Strategy, which emphasize an increase in women’s asset ownership and expansion of their access to formalized bank accounts [142]. Since 2001, the GOU has worked to increase the number of adults accessing formal financial services from 28% in 2009 to 54% in 2013 [143]. With new aims for 70% financial inclusion in the formal financial system by 2017 [144], there is ample opportunity to link the unbanked with national financial institutions, all beginning to use innovative delivery mechanisms to reach the financially excluded (e.g. WESW) [143]. In addition to financial services, skills development is of great interest to the GOU evidenced by its commitment to both formal and non-formal training for new entrants to the labor market in the Business, Technical, Vocational Education and Training (BTVET) Act of 2008 followed by the ten-year BTVET strategic plan (2012–2022). The increasing demand for skills development, particularly among Ugandan youth, has resulted in government expansion of training opportunities [141]. Foundations, and bi-lateral development assistance, have also affirmed the need for greater skills development, initiating supplementary programs. Findings will be disseminated through local, national, and global meetings and publications to facilitate sustainability and scalability.

Our study is supported by extant literature on contingency management, contracting, and voucher systems for vulnerable and stigmatized populations, all of which aim to increase access to income and participation in treatment services and promote engagement in safe and empowering forms of employment among individuals [145, 146]. Our study follows the same underlying premise that when WESW have access to alternative forms of employment and start earning formal income, they may become motivated to improve their skills and employability for professional advancement. The study findings may advance our understanding of how best to implement gender-specific HIV prevention globally [36, 42], engaging women across the HIV treatment cascade (from negative to positive). Further, results will provide evidence for the intervention’s efficacy to reduce STIs and inform implementation sustainability, including costs and cost-effectiveness.

## Data Availability

Not applicable.

## Abbreviations

AE: Adverse event
BTVET: Business, Technical, Vocational Education and Training
CCB: Community collaborative board
CI: Confidence intervals
DFCU: Development Finance Company of Uganda Bank
DSMB: Data safety and monitoring board
DSMP: Data safety and monitoring plan
DTB: Diamond Trust Bank
FL: Financial literacy
GEE: Generalized estimating equations
GOU: Government of Uganda
HIPAA: Health Insurance Portability and Accountability Act of 1996
HIVRR: HIV risk reduction
ICER: Incremental cost-effectiveness ratio
ICHAD: International Center for Child Health and Development
IDA: Individual development account
IRB: Institutional Review Board
MAR: Missing-at-random
MI: Multiple imputation
MPI: Multiple principal investigator
NGO: Non-governmental organizations
PI: Principal investigator
PrEP: Pre-Exposure Prophylaxis
QA: Quality assurance
RA: Research assistant
RBA: Risk Behavior Assessment
RCT: Randomized controlled trial
RHSP: Rakai Health Sciences Program
RTY: Reach the Youth
S: Savings
SAE: Serious adverse event
SSA: Sub-Saharan Africa
STI: Sexually transmitted infection
UGX: Uganda Shillings
UNCST: Uganda National Council of Science and Technology
US: United States
UVRI: Uganda Virus Research Institute
V: Vocational skills training and mentorship
WESW: Women engaged in sex work
